# No evidence of avian influenza antibodies in two species of raptor nestlings inhabiting Norway

**DOI:** 10.1186/s12917-019-2133-0

**Published:** 2019-10-28

**Authors:** Megan Marie Lee, Veerle L. B. Jaspers, Mari E. Løseth, Nathalie Briels, Torgeir Nygård, Jan Ove Bustnes, Courtney A. Waugh

**Affiliations:** 1 Department of Biology, Norwegian University of Science and Technology, Høgskoleringen 5, NO-7491 Trondheim, Norway; 2 Biological Sciences Program, Goucher College, 1021 Dulaney Valley Road, Baltimore, MD 21204 USA; 3 Norwegian Institute for Nature Research, Høgskoleringen 9, 7034 Trondheim, Norway; 4 Faculty of Biosciences and Aquaculture, Nord University, Steinkjer, Norway

**Keywords:** Avian influenza, Birds of prey, Norway, Epizootic event

## Abstract

**Background:**

Since 2016, incursions of highly pathogenic avian influenza virus (HPAIV) H5N8 clade 2.3.4.4b have caused unprecedented clinical signs and mortality in white-tailed eagles (WTE; *Haliaeetus albicilla*) across Europe and have been found to be infecting other raptor species, such as the northern goshawk (NG; *Accipiter gentilis*). Before this study, no screening of Norwegian raptors had been undertaken.

**Results:**

Plasma samples from 43 white-tailed eagle and 29 northern goshawk nestlings, from several locations across Norway were screened for antibodies to avian influenza viruses. No antibodies, and thus, no evidence of AIV exposure, were found in these Norwegian raptors. No clinical signs of AIV were observed in 43 white tailed eagles and 29 northern goshawks.

**Conclusions:**

There are currently no indications that white-tailed eagles and northern goshawks inhabiting Norway are threatened by the recent HPAIV outbreaks in other areas of Europe. Ongoing monitoring should, however, be maintained to detect potential future outbreaks.

## Background

Avian influenza viruses (AIVs) have been isolated from most major families of wild birds worldwide [6] including raptors. In 2016, an unprecedented epizootic event began across Europe caused by a novel reassortment of highly-pathogenic AIVs introduced via migrating birds [1–4]. Infections were reported in 14 European countries [4], with outbreaks in Germany and the Netherlands causing notably more severe symptoms and mortality than previous AIV incidents [2, 3]. In the Netherlands, roughly 13,600 birds spanning 71 species were reported dead, [2]. While the outbreak primarily affected wild aquatic birds, mortality was also observed in raptors, *n* = 158 raptors in the Netherlands, and *n* = 14 raptors in Germany [1, 2, 4].

Raptors appear to be particularly sensitive to this new strain of AIV. As top predators, raptors may be exposed a variety of pathogens present in their prey. Species such as the white-tailed eagle *(Haliaeetus albicilla,)* feed on the carrion of waterfowl [5], which are the primary reservoir for AIV [6]. Raptors may develop immunity to pathogens they are frequently exposed to via prey [1] and mortality in carrion-scavenging raptors can indicate a recent introduction of a pathogen strain to a local avian population [4]. Between November 2016 and April 2017, H5N8 was found to be causing severe clinical signs (neurological signs including torticollis, opisthotonus, ataxia and circling) with 80% mortality of infected white-tailed eagles in northern Germany [1]. Further, in the spring of 2018, raptor species (including white-tailed eagles and northern goshawks amongst others) constituted 74% of the wild birds infected with AIV that were found dead in Europe [7]. The recommendations from these studies [1, 7] were that raptors can act as sentinels for the presence of HPAIV in waterfowl in their range, and that this virus is a new threat to raptors across Eurasia, and thus further biomonitoring across Europe and surrounding areas is required.

Norway has the longest coastline in Europe and is located along the East-Atlantic flyway for migratory birds [8] providing a possible route for exposure of AIVs, such as H5N8, to raptors. Earlier strains of AIV have already been detected in mainland Norway, in gulls and dabbling ducks between 2005 and 2010 [9, 10], and more recently (2017) antibodies against influenza A were detected in gulls inhabiting the Norwegian Arctic region of Svalbard [11]. Earlier strains have also been screened for in Swedish raptor nestling, without any evidence of infection being found [12].

Previous surveys of AIV in Norway have focused on aquatic birds such as ducks, geese, and gulls [10, 13, 14]. One such study found a higher prevalence of AIV in Norwegian wild birds as compared to surveys conducted in other European countries [13]. AIV is more stable in water at cooler temperatures [15] and thus Norway’s cold climate may facilitate increased environmental persistence [10] and increased transmission rates.

For these reasons, as well as the lack of historical data on AIV prevalence in Norwegian raptor species, we screened for avian influenza antibodies in 43 white-tailed eagle and 29 northern goshawk (NG; *Accipiter gentilis*) nestlings from several locations across Norway.

Maternal antibody transmission of AIV has been well documented in birds (e.g. yellow-legged gulls [16], ring-billed gulls [17], and mallards [18]). Thus, due to the logistical and ethical benefits of sampling nestlings over adults, monitoring antibodies in nestlings has been proposed as a key tool to monitor disease in adult raptors [19].

Thus, the discovery of AIV antibodies in a raptor nestling up to 4 weeks of age, would indicate the presence of circulating antibodies in the mother bird.

Currently, data on AIV in Norwegian raptor species are non-existent. The present study thus aimed to provide important baseline data on the occurrence of AIV by sampling during a temporally-relevant period the H5N8 European epizootic in 2016.

## Results

In this study, plasma from 43 Norwegian white-tailed eagles and 29 northern goshawk nestlings was screened for AIV antibodies as an indicator of circulating AIV in these populations (see Table 1 for sampling details). No antibodies were found in any samples from any location. There were no clinical signs of disease (i.e. neurological signs including torticollis, opisthotonus, ataxia and circling) that could be associated with AIV. Nestling body weights (white tailed eagle: mean ± sd = 4.99 ± 0.66 kg, goshawk: 0.87 ± 0.20 kg) indicated that the investigated populations in Norway currently show no specific health issues.

**Table 1:** Overview of sampling locations and sample sizes for plasma taken from Norwegian raptor nestlings in 2016 and screened for AIV antibodies in this study

Species	Location	*n*
White-Tailed Eagle	Steigen	21
White-Tailed Eagle	Smøla	22
Northern Goshawk	Trøndelag	19
Northern Goshawk	Troms	10

## Discussion

HPAIV infections have been reported to cause disease or mortality in white-tailed eagles and northern goshawks across other regions of Europe (e.g .[3, 7]). A recent example highlights the virulence of these HPAIV infections, showing an 80% mortality rate in infected white-tailed eagles in Germany over the winter of 2016/2017 [1].

**Fig. 1:**
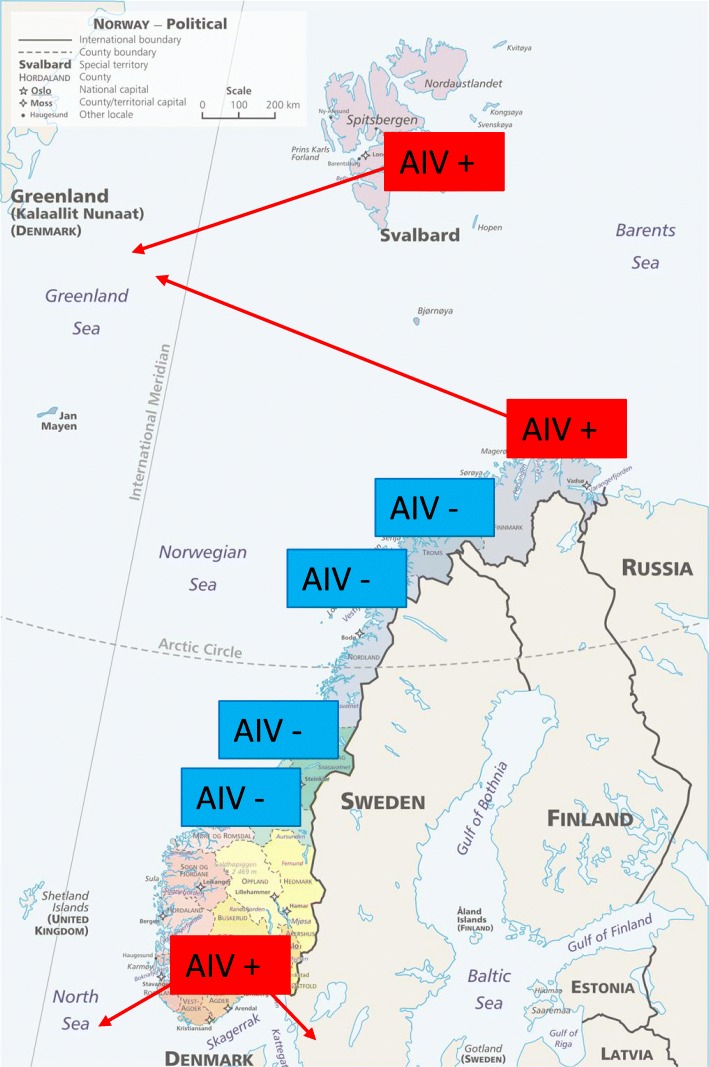
Map of the screening locations of Norwegian wild birds for avian influenza (AIV). Red indicates wild birds positive for AIV and green indicates negative for AIV. Previous data was taken from Tønnessen et al. [9] (northern Norway) and Kulberg Sjurseth et al. [20] (middle to southern Norway). WTE: white-tailed eagle (*n* = 43); NG: northern goshawk (*n* = 29) are from the current study. Red lines indicate the predicted migration routes using data and information from Norwegian SEATRACK project, www.seapop.no/en/seatrack and Dalby et al. [24]

Incursions of AIV into Norwegian populations of raptors have yet to be reported. All current and ongoing AIV screening in wildlife in Norway is restricted to waterfowl and gulls, and as of 2016, only low pathogenic avian influenza strains had been detected [9, 20] Fig. 1).

This study is the first study to screen Norwegian raptors for seroconversion and report negative findings (Fig. 1). Further evidence that these populations are currently AIV-free are that we reported no AIV associated clinical disease in these nestlings nor in the adults tending to the nestlings. A recent study has also looked at the protein fractions in the plasma of the same WTE nestlings, and found that all levels were well withint he rang of normal for healthy raptor nestling, with no indications of infections [21].

The use of nestlings has been proposed as a key tool for assessing disease status in adults. A previous study of maternal antibody transfer in raptors, showed that while both the prevalence and titers of antibodies were lower in chicks than in adults [19], nestlings still would provide an indication that the infection was prevalent in the population or not. Thereby, if a disease is endemic in a population, we would expect to detect antibodies in the nestlings, jus at a lower prevalence and titre than in adults. AIV maternal antibodies persist in chicks for 4–5 weeks [22]. Therefore in our current study northern goshawks provide an indication of the adults via maternal transmission (antibodies and/or virus), whereas white tailed eagles (that were samples 11 weeks after hatching) provide an indication of other methods of exposure (i.e. through the diet).

The locations of previous AIV detections in Norway may also explain the lack of AIV antibodies discovered in this study. While AIV has been screened for across mainland Norway, it has only been detected in southern regions below 60°N and in northern regions above 70°N (e.g. Hornøya, 70°22′ N). There is currently no AIV being detected in the middle regions between circa 60° and 70 N. (Fig. 1). AIV is spread via the migration route of birds. The birds (such as gulls and waterfowl) that are AIV-positive in Norway have migration routes that either come in from the south, or from the north (Norwegian SEATRACK project, www.seapop.no/en/seatrack; [24]), thus explaining why the middle regions are infection-free. However, as there are populations of seabirds and waterfowl that inhabit these middle areas of Norway, and share over-wintering grounds with the infected populations (Norwegian SEATRACK project, www.seapop.no/en/seatrack) we can predict that AIV infections will become present in these areas in the future. As the distributions and migration patterns of these avian species that carry AIV are predicted to shift in response to climate change, the dynamics of the disease are also likely to be affected. This may also result in new disease challenges for avian species, including raptors.

## Conclusions

No antibodies were found in any plasma sample of white-tailed eagles or northern goshawks from the studied locations within Norway. Thus, no evidence has yet been found of AIV exposure in Norwegian white-tailed eagle or northern goshawk nestlings. Ongoing monitoring of these species is recommended as it would provide an early warning system for the arrival of HPAIV into these populations and allow for mitigation measures before significant mortalities arise.

## Methods

For each species, plasma samples were obtained from wild populations across Norway in 2016 (Table 1). Free living white-tailed eagle nestlings were sampled at Steigen and Smøla at approximately 10 ± 1.5 weeks of age, as described in detail previously [21]. Free-living northern goshawks, between 3 to 5 weeks of age, were sampled in Troms and Trøndelag as described in detail previously [23]. Samples were taken from single nestlings in independent nests (Table 1). C. The sampling was approved by the Norwegian Food Safety Authority (Mattilsynet 2016/8709) and the handling of the birds were in accordance with the regulations of the Norwegian Animal Welfare Act.

A pan species Influenza A virus antibody test kit (IDEXX) was used to screen goshawk (*n* = 29) and eagle (*n* = 43) plasma aliquots. Absorbance values were measured with a Cytation 5 Imaging Reader (BioTek).

Nestlings were observed for any signs of clinical disease associated with AIV, or other disease, such as torticollis and coordination problems, i.e. wings are dropped and the raptor is crouching on its intertarsal joints.

## Data Availability

The datasets used and/or analysed during the current study available from the corresponding author on reasonable request.

## Abbreviations

AIVs: Avian influenza viruses
HPAIV: Highly pathogenic avian influenza virus
NG: Northern goshawk
WTEs: White-tailed eagles
